# Bioactivity Studies on Titania Coatings and the Estimation of Their Usefulness in the Modification of Implant Surfaces

**DOI:** 10.3390/nano7040090

**Published:** 2017-04-22

**Authors:** Aleksandra Radtke, Adrian Topolski, Tomasz Jędrzejewski, Wiesław Kozak, Beata Sadowska, Marzena Więckowska-Szakiel, Piotr Piszczek

**Affiliations:** 1 Faculty of Chemistry, Nicolaus Copernicus University in Toruń, Gagarina 7, 87-100 Toruń, Poland; topolski@umk.pl (A.T.); piszczek@chem.umk.pl (P.P.); 2 Nano-Implant Ltd. Gagarina 5, 87-100 Toruń, Poland; 3 Faculty of Biology and Environmental Protection, Nicolaus Copernicus University in Toruń, Lwowska 1, 87-100 Toruń, Poland; tomaszjedrzejewski1983@gmail.com (T.J.); wkozak@umk.pl (W.K.); 4 Laboratory of Infectious Biology, Faculty of Biology and Environmental Protection, University of Lodz, Banacha 12/16, 90-237 Łódź, Poland; beata.sadowska@biol.uni.lodz.pl (B.S.); marzena.wieckowska@biol.uni.lodz.pl (M.W.-S.)

**Keywords:** titania, nanofibers, nanowires, thermal oxidation, Ti6Al4V

## Abstract

Morphologically different titania coatings (nanofibers (TNFs), nanoneedles (TNNs), and nanowires (TNWs)) were studied as potential biomedical materials. The abovementioned systems were produced in situ on Ti6Al4V substrates via direct oxidation processes using H_2_O_2_ and H_2_O_2_/CaCl_2_ agents, and via thermal oxidation in the presence of Ar and Ar/H_2_O_2_. X-ray diffraction and Raman spectroscopy have been used to structurally characterize the produced materials. The morphology changes on the titanium alloy surface were investigated using scanning electron microscopy. The bioactivity of the samples has been estimated by the analysis of the produced titania coatings’ biocompatibility, and by the determination of their ability to reduce bacterial biofilm formation. The photoactivity of the produced nanocoatings was also analyzed, in order to determine the possibility of using titania coated implant surfaces in the sterilization process of implants. Photocatalytic activity was estimated using the methylene blue photodegradation kinetics, in the presence of UV light.

## 1. Introduction

The functional connection between the bone tissue and the implant surface (osseointegration) is considered as the main prerequisite for the long-term clinical success of the implantation procedure [1,2,3,4,5]. The structure, morphology, and wettability of the implant surface play a key role in osseointegration processes [6,7,8,9,10]. Titanium and its alloys are widely used materials in the construction of implants utilized in orthopaedics and in maxillofacial surgery, however these materials do not form direct connections with the living bone [11,12,13,14]. Therefore, surface modifications are the most important methods used for osseointegration enhancement [15,16,17]. Recent efforts in this field have highlighted the importance of nanotechnology in altering the surface morphology to better mimic the surface roughness features of the natural bone and to favour the positive interaction with cells [18,19,20]. According to earlier reports, the materials based on titanium dioxide are especially important for surface modification, due to their good osseointegration properties, high corrosion resistance, and thermal stability [21,22,23]. Titania materials of different 1-D nanoarchitectures, such as nanowires (TNWs), nanotubes (TNTs), and nanofibers (TNFs), have been intensively studied worldwide due to their potential technological applications in many areas including the next generation of implant production [24,25,26,27,28,29,30,31,32,33,34]. Titania nanotube layers are especially intensively studied due to their facile synthesis, the ability to control the nanotube parameters (e.g., their diameter, length, and structure), and their enhanced cellular responses. This enhancement of the positive cellular behaviour was proven with the use of different types of cells, such as fibroblasts, osteoblasts, chondrocytes, endothelial cells, and mesenchymal stem cells [35,36,37,38,39,40]. The applications of titania nanofibrous coatings and nanowires as potential bioactive mediums, which can be formed on the surface of implants, have been intensively studied in last years. These materials are characterized by high porosity, high surface to volume ratio, and morphology which is similar to the natural extra-cellular matrix [41,42,43,44,45,46,47,48]. Analysis of previous reports showed that TNFs and TNWs have been produced using the electrospinning method, laser ablation, anodization, hydrothermal treatment, and gas phase reactions [49]. Taking into account the fact that the widespread utilization of nanostructured TiO_2_ is often hindered by the opposite demands for the precise control of well-ordered surface features and low-cost, energy friendly, and fast production, in our investigations we have used very simple methods based on direct chemical oxidation and thermal oxidation of titanium alloy substrates. We have focused on the determination of the adsorption and proliferation of fibroblast cells on nanostructured titania surfaces, and on the characterization of the antimicrobial activity of these coatings. 

In this paper we have characterized the biological activity of nanofibrous titania coatings, TiO_2_ nanoneedles and nanowires, and compared them with the activity of Ti6Al4V, which is commonly used in the construction of implants. Our goal is to optimize the nanofiber and nanowire manufacturing process with the use of a very simple procedure of direct Ti6Al4V oxidation in solution and its thermal oxidation, in possibly low temperatures. Moreover, we assumed that the studied TiO_2_ nanoarchitectures will have an influence on the photoactivity of the Ti6Al4V surface. Such modified surface would be possible to be disinfected/sterilized with the use of UV light. It is an important property because the same coating could play a dual role for the implants—increase the osseointegration process and create optimal conditions for carrying out the process of implant surface sterilization/disinfection with the use of UV light. 

## 2. Results

### 2.1. The Production of Titania Nanofibrous Coatings (TNFs) and Their Characterization

Titania based coatings were produced on the surface of Ti6Al4V substrates by direct oxidation, using 30% H_2_O_2_ and H_2_O_2_/CaCl_2_ solutions as oxidizing agents. The oxidation process was conducted at 80 and 100 °C, and the time of oxidation was 6, 8, 10, and 12 h respectively. The chosen method led to the production of nanofibrous layers with different morphologies, depending on the temperature and the oxidation time. On the basis of the preliminary SEM studies of the coatings, the following samples have been chosen for further biological experiments and the following names have been given to the systems: coatings obtained by direct oxidation, using 30% H_2_O_2_ (temperature [°C], time of process [h]): (80, 8)–TNF1, (100, 8)–TNF2, (100, 12)–TNF3, and coatings obtained by direct oxidation with the use of H_2_O_2_/CaCl_2_ (temperature [°C], time of process [h]): (80, 8)–TNF4, (100, 8)–TNF5, (100, 12)–TNF6. According to the GIXRD (grazing incidence X-ray diffraction) and Raman spectroscopy studies, it can be stated that all obtained fibrous materials were amorphous. However, the analysis of the Raman microscopy data revealed that the presence of anatase nanograins on the surface of some samples obtained by the oxidation using H_2_O_2_ is also possible. The very weak bands, which were detected at around 396, 513, and 637 cm^−1^ in the Raman spectra at selected places on the sample surface, confirm this. 

**Figure 1:**
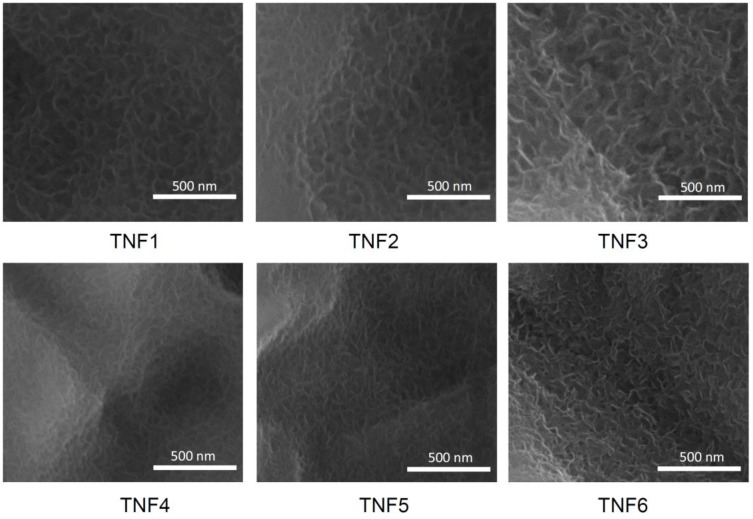
Scanning electron microscopy (SEM) images of titania nanofibers (TNF, produced by the direct oxidation of Ti6Al4V substrates, using H_2_O_2_ (TNF1, TNF2, TNF3) and H_2_O_2_/CaCl_2_ agents (TNF4, TNF5, TNF6).

The formation of TNF coatings during the Ti6Al4V oxidation with H_2_O_2_ and layers containing TiO_2_ and 0.32–0.51 mass % of calcium atoms (obtained from the H_2_O_2_/CaCl_2_ oxidation mixture) was confirmed by the SEM and EDX investigations. Analysis of SEM images obtained for the samples produced at 8 and 12 h showed the uniform surface coverage by the produced TNF coatings without cracks and gaps (Figure 1). According to these data, we can state that independent of the oxidation temperature, increasing the oxidation time has a direct impact on the quality of the nanofibers. Moreover, layers produced with the use of H_2_O_2_/CaCl_2_ are characterized by thinner fibers (~13–15 nm), in the comparison to those obtained in the same oxidation conditions (temperature, time) but with the use of H_2_O_2_ (~22–25 nm). 

### 2.2. Titania Coatings Produced by Thermal Oxidation and Their Characterization

**Figure 2:**
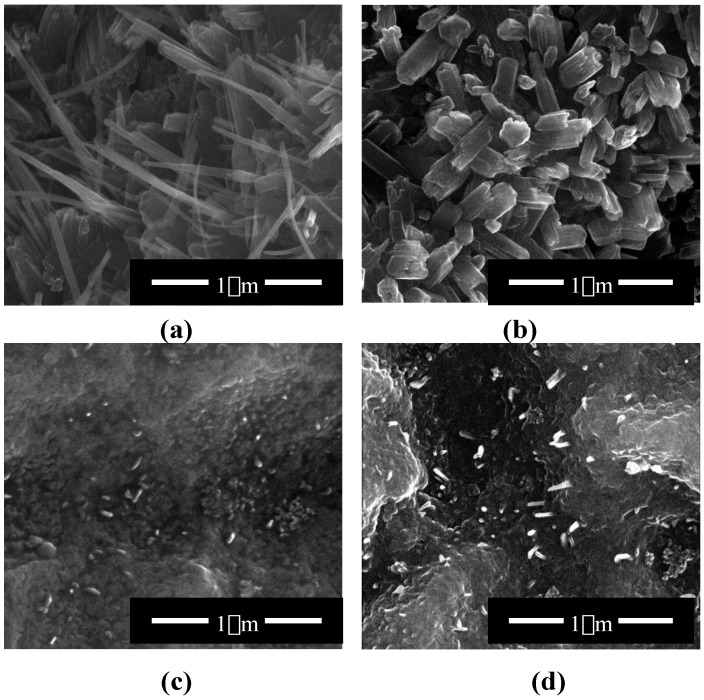
SEM images of titania nanoneedles (TNN3, (**a**)), titania facetted crystals (TNN2 (**b**)), and titania nanowires (TNW1 (**c**)) and (TNW6 (**d**)).

The thermal oxidation of Ti6Al4V substrates was carried out in the presence of Ar or Ar/H_2_O_2_ (flow rates: 30 and 100 cm^3^/min) in the temperature range from 475 to 500 °C. Analysis of the fibrous coatings by SEM showed significant differences in the morphology of the produced coatings on the surface of the Ti6Al4V substrates, during thermal oxidation with the use of Ar and Ar/H_2_O_2_. Coatings formed in the presence of pure argon, using a low argon flow rate (30 cm^3^/min) and temperatures between 475 and 500 °C, were composed of dense packed and morphologically well oriented nanowires, with nanoneedle (TNNs) morphology (Figure 2a). The following names have been given for better clarity to the systems produced in Ar atmosphere (flow rate [cm^3^/min], temperature [°C], time of process [h]): (30, 500, 2)–TNN1, (100, 500, 2)–TNN2, (30, 475, 2)–TNN3, (100, 475, 2)–TNN4, (30, 500, 6)–TNN5, (100, 500, 6)–TNN6, (30, 475, 6)–TNN7, and (100, 475, 6)–TNN8. The increase of the oxidation temperature to 500 °C while maintaining the same gas flow led to the formation of the coating composed of faceted crystals (Figure 2b). The use of Ar/H_2_O_2_ as the oxidizing medium led to the nanotextured surface with dispersed nanowires (Figure 2c). The formation of TNWs was much slower and less efficient in the presence of Ar/H_2_O_2_, completing the oxidation in 2 h. The prolongation of the process time improved the quality of the nanowires, but only slightly, and failed to obtain a system similar to those received in the presence of pure argon (Figure 2d). The following names have been given for better clarity to systems produced in Ar/H_2_O_2_ atmosphere (flow rate [cm^3^/min], temperature [°C], time of process [h]) (30, 500, 2)–TNW1, (100, 500, 2)–TNW2, (30, 475, 2)–TNW3, (100, 475, 2)–TNW4, (30, 500, 6)–TNW5, (100, 500, 6)–TNW6, (30, 475, 6)–TNW7, and (100, 475, 6)–TNW8. Differences in the morphology of the TiO_2_ nanostructures are the consequence of the Ti6Al4V thermal oxidation mechanism, which is based on the well-known Deal-Grove model [50]. According to this model mechanism, at the early stages of oxidation, the initial oxide layer is formed within the oxidation reaction. When the oxide layer becomes thicker, the timescale of diffusion through this layer transcends the oxidation reaction timescale at the Ti-TiO_2_ interface, and the nanowire formation becomes diffusion limited [50].

**Figure 3:**
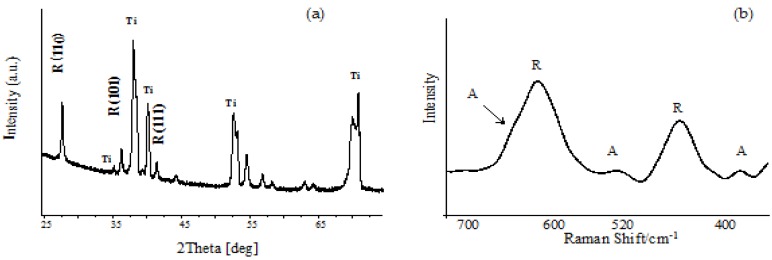
Grazing incidence X-ray diffraction (GIXRD) spectrum (**a**) and Raman spectrum (**b**) of titania nanowires (TNW4) obtained during the thermal oxidation of Ti6Al4V with the use of Ar/H_2_O_2_, *T* = 500 °C.

The structure of the produced titania layers was determined using GIXRD (grazing incidence X-ray diffraction) and Raman spectroscopy (Figure 3). Results of the GIXRD studies of TNN and TNW proved the formation of only the TiO_2_ rutile form (the lack of 25.33 (101), 37.80 (004), 48.08 (200), 55.12 (211) signals, Figure 3a). Analysis of the Raman spectra confirmed the formation of the TiO_2_ rutile form during the thermal oxidation of Ti6Al4V substrates in the presence of pure argon, using a low argon flow rate (30 cm^3^/min) and a temperature region of 475–500 °C (TNN coatings). Simultaneously, the analysis of the Raman spectra of the TNW samples (the use of Ar/H_2_O_2_ oxidation factor), between 300 and 750 cm^−1^, revealed the presence of weak or very weak bands at 325, 520, and 640 cm^−1^ (the weak band at 640 cm^−1^ overlaps the strong band at 612 cm^−1^ of the rutile form) which indicated the presence of small, dispersed anatase grains (Figure 3b). The presence of anatase nanograins in the structure of the TNW materials can be explained by the incomplete transition of the initial anatase grains to rutile phase in the temperature range from 475 to 500 °C. However, intensity of the bands assigned to the rutile form indicates that it is the dominant form.

Taking into account that rutile crystals exhibit a prismatic or acicular growth habit with preferential orientation along their *c*-axis, as the {110} facets of rutile crystals exhibit the lowest surface free energy and are therefore thermodynamically most stable, we can state that the *c*-axis oriented growth of rutile crystal appears clearly in our TNN samples, as is visible in Figure 2a,b [51,52]. Another growth habit in the form of abnormal grain growth phenomena of the rutile phase, also referred to exaggerated or secondary recrystallization grain growth, is visible for the TNW coatings (Figure 2c,d). We have noticed a grain growth phenomenon for TNW through which certain energetically favorable crystallites grow rapidly in a matrix of finer grains resulting in a bimodal grain size distribution [53].

### 2.3. Fibroblasts’ Adhesion and Proliferation

**Figure 4:**
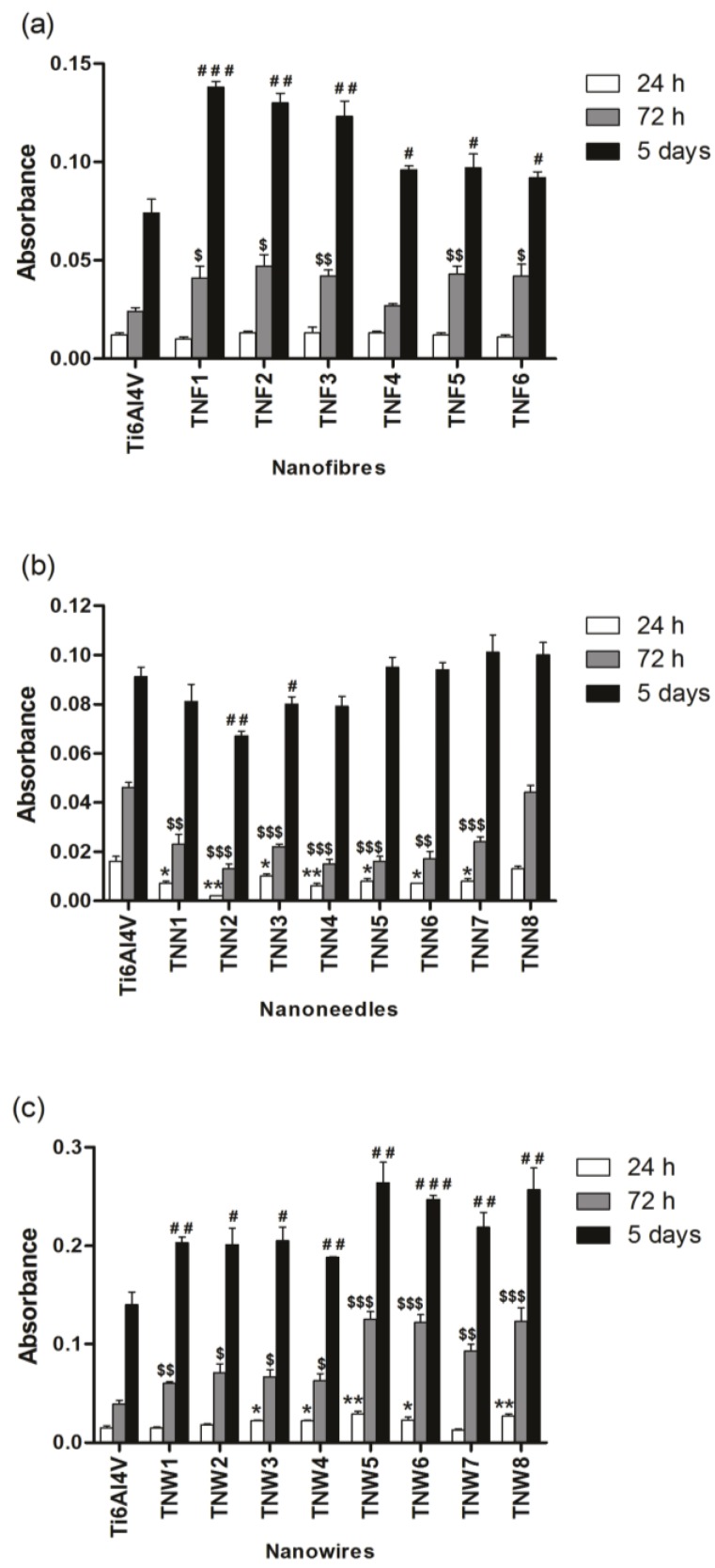
Effect of incubation time (24 h, 72 h, and 5 days, respectively) on the murine L929 fibroblasts’ adhesion (after 24 h) and proliferation (after 72 h and 5 days) on the surfaces of titania nanofibers (TNF; (**a**)), nanoneedles (TNN; (**b**)) and nanowires (TNW; (**c**)) detected by the MTT assay. Data revealed that with an increase of incubation time, more L929 cells proliferated on all of the specimens of nanofibers as well as on the nanowires arrays, and this phenomenon did not occur in the case of nanoneedles. Cell viability was assessed by an MTT assay for the 24 h, 72 h, and 5 day incubation time with the respective samples. The results are shown as the mean of absorbance values ± standard error (S.E.) of three experiments. The asterisk indicates significant differences in the level of adhesion (after 24 h) observed for the respective titania alloy reference samples (Ti6Al4V) compared to the tested TNF, TNN, or TNW (* *p* < 0.05, ** *p* < 0.01). ‘$’ denotes significant differences in the proliferation level after 72 h noticed for the respective Ti6Al4V in comparison to the tested nanolayers (^$^ *p* < 0.05; ^$$^ *p* < 0.01; ^$$$^ *p* < 0.001). The hash mark indicates significant differences between the cells incubated on the respective Ti6Al4V and the fibroblasts incubated on the surface of the tested TNF, TNN, or TNW for 5 days (^#^ < *p* < 0.05; ^# #^ *p* < 0.01, ^# # #^ *p* < 0.001).

Figure 4 presents the influence of the surface morphology (TNFs–Figure 4a, TNNs–Figure 4b, and TNWs–Figure 4c) on the L929 murine fibroblasts’ adhesion (after 24 h) and proliferation (after 72 h and 5 days), which were evaluated based on the results of the MTT (3-(4,5-dimethylthiazol-2-yl)-2,5-diphenyltetrazolium bromide) assay. Analysis of these data revealed that with an increase of incubation time, more L929 cells proliferated on all of the specimens of nanofibers as well as nanowire arrays. This phenomenon was particularly noticeable after the 5 days incubation time. Significantly, the level of proliferation was much greater on the surface of the tested TNFs and TNWs specimens in comparison to the appropriate titania alloy reference samples (Ti6Al4V). According to the data presented in Figure 4, the highest level of the proliferation was observed for titania nanofibrous coatings produced by the Ti6Al4V oxidation using H_2_O_2_ (TNF1, TNF2, TNF3), which was especially noticeable for the 5 days incubation time. Simultaneously, we did not notice any differences in the level of adhesion (after 24 h) of cells incubated with the tested nanofibers, compared to the reference sample. In the case of nanowires, studies showed that the highest level of increase in the fibroblasts’ proliferation was achieved for the nanowires obtained during 6 h of the oxidation process (TNW5–TNW8). It was observed both after 72 h as well as after the 5 days incubation time (Figure 4c). 

Completely different behaviour was noticed for the titania nanoneedles’ (TNNs) surface. As it is shown in Figure 4b, the level of cell adhesion (after 24 h) was lower in all the studied TNNs samples, except TNN8, in comparison to Ti6Al4V. A similar effect was observed in the case of the fibroblasts’ proliferation measured after 72 h. Finally the proliferation level observed after 5 days was for all studied TNNs comparable or lower than that for the reference sample. This kind of morphology, even if it is a phenomenon from the nanoarchitecture point of view, is not optimal for the fibroblasts’ adhesion and proliferation. The most important property of the surface, which can be used as the implant surface coating, is its biocompatibility and the ability to promote fibroblasts’ adhesion and proliferation enhancement. Without this attribute, there is no chance to use such coatings in implantology. For this reason, the TNNs were not subjected to further microbiological tests and studies.

**Figure 5:**
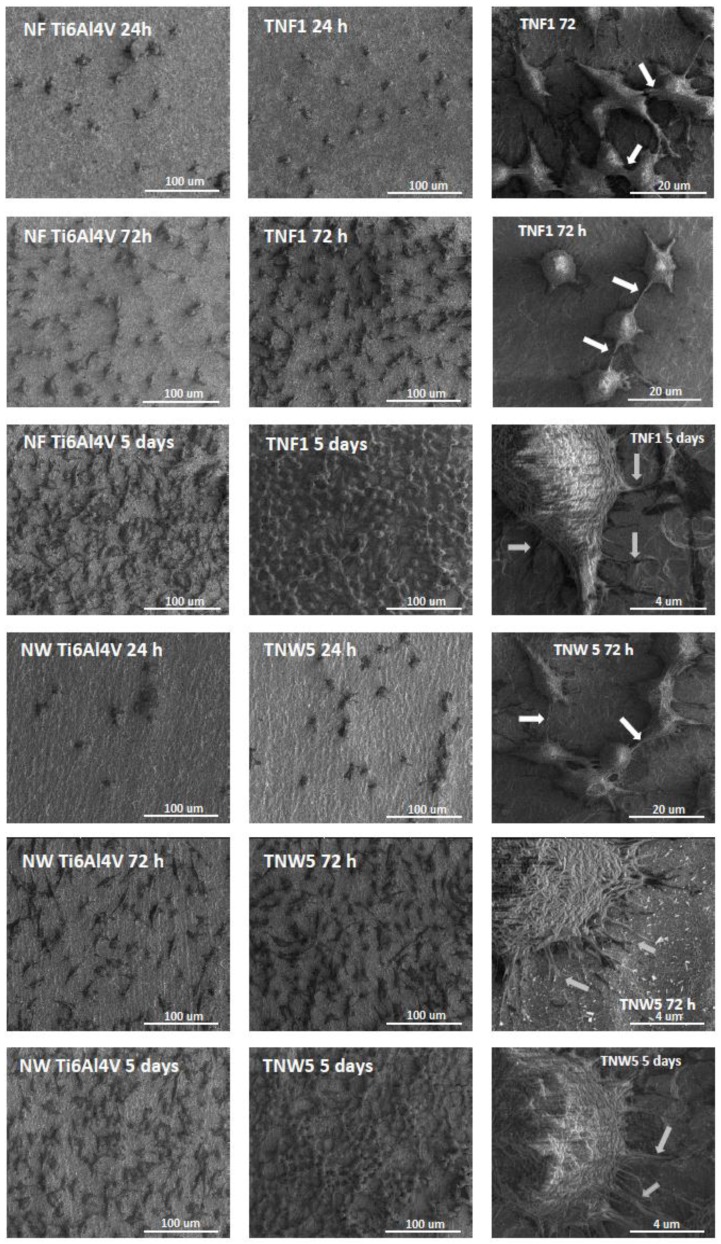
Scanning electron microscopy (SEM) images showing the murine L929 fibroblasts’ adhesion (after 24 h) and proliferation (after 72 h or 5 days) on the surfaces of the tested titania nanofibers 1 (TNF1) and nanowires 5 (TNW5) compared to the titania alloy reference samples (NF Ti6Al4V and NW Ti6Al4V, respectively). The results clearly indicate the high level of biocompatibility of the tested biomaterials, which is confirmed by the increase in the number of cells attached to the surface of the nanomaterials over time and the formation of filopodia by the L929 cells. The white arrows in the figure indicate the filopodia spread between the fibroblast cells, whereas the grey arrows present filopodia penetrating deep into the nanolayers.

Figure 5 shows the comparative scanning electron microscopy (SEM) micrographs of L929 murine fibroblasts cultured on one of the studied titania nanofiber coatings (TNF1) and one of the titania nanowire layers (TNW5) for 24 h, 72 h, or 5 days in comparison to the respective reference samples. Our results from the SEM analysis clearly indicate the high level of biocompatibility of the tested biomaterials supporting the MTT results. It is worth noting that a favourable cellular interaction with the biomaterial surface is critical for the long-term success of the implants [54].

### 2.4. The Microbial Aggregates/Biofilm Formation on Titania Coatings

**Figure 6:**
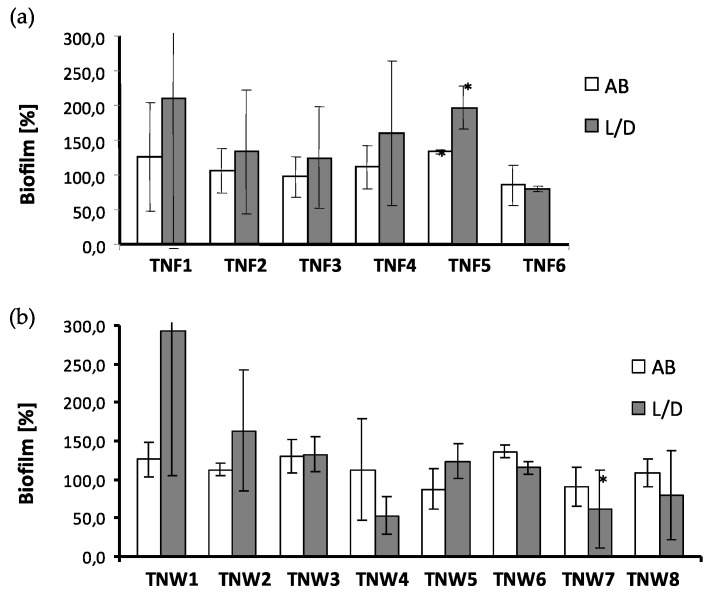
*S. aureus* ATCC 29213 aggregates/biofilm formation (after 24 h) on the surfaces of titania nanofibers (TNF; **a**) and nanowires (TNW; **b**) detected by the Alamar Blue assay and the Live/Dead BacLight Bacterial Viability kit (alive bacteria). The results are provided as the mean percentage ± standard deviation (SD) of staphylococcal aggregates/biofilm formation compared to the colonization of the reference sample (Ti6Al4V) considered as 100%. *p* < 0.05 was considered significant. Two independent sets of experiments with four replicates in each were performed.

Titania nanofiber (TNFs) and nanowire (TNWs) samples were exposed for 24 h on staphylococci to assess their vulnerability to bacterial colonization. The presence of *S. aureus* ATCC 29213 aggregates/biofilm on the surface of TNF and TNW samples was tested, using two independent methods: the Alamar Blue assay (AB) showing only metabolically active cells, and the Live/Dead BacLight Bacterial Viability kit (L/D) indicating alive and/or dead microorganisms. The bbtained results are presented in Figure 6a,b for the TNFs and TNWs, respectively.

Because of the different phases of microbial cells (especially those forming biofilms) in terms of their metabolic activity and viability (alive metabolically active cells, alive dormant cells, dead cells), differences in the results for each sample between both methods may occur. In the case of nanofiber coatings, only TNF6 was able to reduce staphylococcal aggregates/biofilm formation in comparison to the Ti6Al4V on which the developed microbial biofilm was considered as 100% (Figure 6a). The average percentage of biofilm inhibition caused by TNF6 was 17.5 ± 24.7% (*p* = 0.6431) and 19.9 ± 3.4% (*p* = 0.0929) in AB and L/D, respectively. The vulnerability to staphylococcal colonization of the biomaterials TNF2–TNF4 was similar to that exhibited by the reference sample. For all of those coatings (TNF2–TNF4, TNF6) the obtained results were advantageous in terms of their possible use in medical implants. The results of staphylococcal aggregates/biofilm formation on the nanowire samples are presented in Figure 6b. The biomaterials labelled as TNW3, TNW5, and TNW6 exhibited similar vulnerability to microbial colonization as the reference sample (Ti6Al4V). The average percentage of *S. aureus* aggregates/biofilm formation tested by both methods reached a maximum of 132.7 ± 23.0% (*p* = 0.0929), 123.8 ± 21.8% (*p* = 0.1152), and 136.1 ± 8.3% (*p* = 0.0929) for the samples mentioned above, respectively. This indicates that the proper balance between tissue biocompatibility of these surfaces and their vulnerability to microbial colonization was preserved. Even more favourable results, with regards to the use of the tested titania samples in implantology, were obtained for TNW4, TNW7, and TNW8. All of these samples demonstrated the ability to inhibit bacterial biofilm formation when assessed by L/D (Figure 6b). The percentage of *S. aureus* biofilm repression was in the range of 31.0%–46.8%, wherein only for TNW7 were the observed differences significant in comparison to Ti6Al4V (*p* = 0.0016). The results obtained for TNW1 and TNW2 are not clear. The process of microbial colonization of these biomaterials was similar as in the case of the reference sample but only when tested by AB (the average percentage of *S. aureus* aggregates/biofilm formation reached 125.8 ± 22.6% (*p* = 0.1152) and 112.4 ± 8.1% (*p* = 0.0927) for TNW1 and TNW2, respectively). When the L/D method was used, much more bacteria were detected on the surface of both of these samples, exceeding 2.9× and 1.6× (for TNW1 and TNW2, respectively) the colonization level of the control Ti6Al4V sample. It is evident that the *S. aureus* adhered to TNW1 and TNW2 were still alive although they did not retain the metabolic activity. It can be speculated that both of these samples possess bacteriostatic activity, though the high level of staphylococcal colonization rather excludes them from the biomaterials suitable for implantation. 

### 2.5. Wettability of TiO_2_ Nanostructures

**Figure 7:**
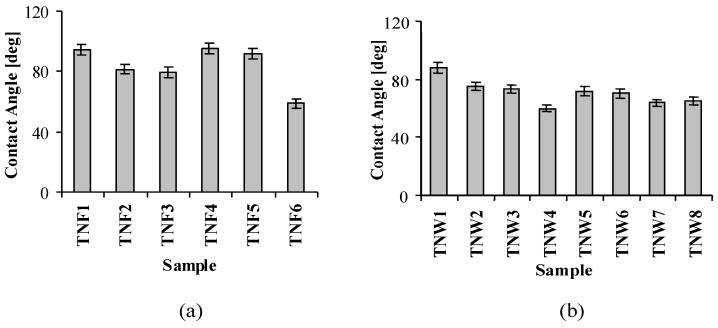
The results of the wettability studies on the TiO_2_ nanofibers (TNF) (**a**), and TiO_2_ nanowires (TNW) (**b**).

The results of the wettability studies on the produced materials showed clear differences in their values of the contact angle (Figure 7a,b). Almost all the studied nanofibrous coatings, except TNF6, are characterized by a rather hydrophobic character (contact angle is in the range 79°–95°). The TNF6 sample, which was produced at 100 °C for 12 h and in the presence of CaCl_2_, shows a much more hydrophilic nature (Figure 7a). The obtained results indicate the clear tendency of the decrease of the contact angle and thereby the increase of the surface hydrophilicity with the increase of the temperature and the time of oxidation. In the case of the TNW coatings, the dependency between the surface hydrophilicity and the argon flow rate was noticed. For the higher Ar flow rate, the surface of TNW coatings is characterized by higher hydrophilicity (Figure 7b). 

### 2.6. Photocatalytic Activity

**Figure 8:**
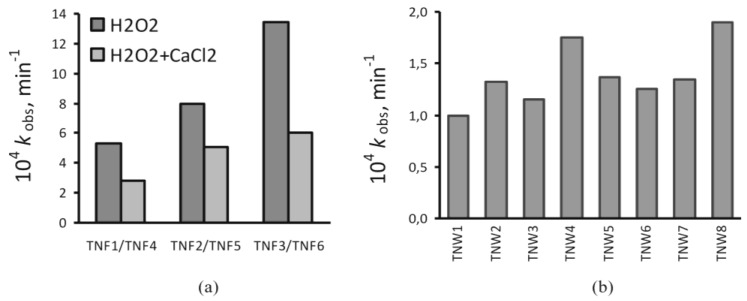
Dependencies of the observed methylene blue photodegradation rate constants of titania nanofibers (**a**) and titania nanowires (**b**); the results take into account blind tests (no UV and no titania substrates).

The photocatalytic properties of the TNF and TNW materials were estimated by studying the photodegradation of methylene blue (MB) under UV irradiation. The degradation rate constant of MB was used to characterize the photocatalytic activity of the produced titania coatings. Figure 8 shows that the values of the rate constant (*k*_obs_) are in the range from 1 to 13 × 10^−4^ min^−1^. This means that the half-times for the studied process are in the range of 8–115 h. Thus, the decomposition of methylene blue is relatively slow in the studied conditions.

The analysis of Figure 8 shows that the TNF coatings produced at higher temperature and at longer oxidation time promote the MB decomposition. This tendency of the observed changes is the same as in the case of TNFs obtained using the H_2_O_2_ and H_2_O_2_/CaCl_2_ oxidation agents. Incorporation of calcium ions into the structure of titania nanofibers (TNF4, TNF5, and TNF6) results in a lower rate of MB photodegradation (Figure 8a). A significantly slower MB photodegradation process was observed for TNWs. Almost all calculated observed rate constants are close to 1.3 × 10^−4^ min^−1^. The lowest reaction rate is noticed for TNW1, whereas the *k*_obs_ values for TNW4 and TNW8 are distinctly higher than for the other nanowire coatings. According to the results presented in Figure 8b, it can be stated that the photoactivity of TNW coatings obtained at 500 °C was lower in comparison to the nanowires formed at 475 °C (e.g., TNW3 vs. TNW1, TNW4 vs. TNW2, TNW7 vs. TNW5, TNW8 vs. TNW6). Moreover, the TNW systems produced at the higher gas flow and during the longer oxidation time revealed better activity in the photodegradation of MB (Figure 8b). Cross-analysis of these data shows that the highest rates of MB photodegradation are for TNWs fabricated at lower temperature (475 °C) with higher gas flow (100 mL/min). 

## 3. Discussion

TiO_2_ coatings of different structures and surface morphologies were produced using two different methods of Ti6Al4V surface oxidation. The chemical oxidation of the substrate surface in the presence of such oxidants as H_2_O_2_ and H_2_O_2_/CaCl_2_ at 80 and 100 °C resulted in the formation of amorphous titania nanofibrous (TNFs) coatings. The presence of calcium ions in the oxidation mixture caused the production of TiO_2_ layers containing 0.32–0.51 mass % of calcium atom additives. Thermal oxidation of Ti6Al4V substrates in the presence of pure argon led to the formation of rutile TNN coatings. The application of Ar/H_2_O_2_ as an oxidation agent caused the production of rutile TNW coatings containing dispersed anatase grains.

The possible application of the studied materials as bioactive coatings required investigations associated with their wettability, biocompatibility, and their ability to reduce bacterial adhesion and biofilm formation. The results of the contact angle measurements indicated that the oxidation of the Ti6Al4V surface did not drastically change the character of this surface (contact angle of Ti6Al4V = 83°), except for TNF6, TNW4, TNW7, and TNW8, for which we expected the clearly different biological activity versus the other produced layers. The comparison of the wettability and photoactivity study results exhibited a distinct correlation between the observed rate constants and contact angle values. For samples, which were characterized by the higher value of the contact angle, the lower rate of MB photodegradation was noticed. The highest values of the contact angle for the TNF1, TNF4, and TNF5 samples and simultaneously the low values of the rate constants are good illustrations of this fact (Figure 8). Similarly, TNW1 possessing the highest contact angle among all the studied TNWs degrades MB the slowest. In contrast, TNW4 and TNW8, which have relatively low contact angles, degrade MB with the highest rate constants. Samples exhibiting the best photoactivity could be optimally subjected to a sterilization process, using UV light, which is an important element of implant preparation before the implantation process.

The biocompatibility research based on the MTT assays proved that the highest level of fibroblast cell proliferation was observed for titania nanofibrous coatings produced by the oxidation using H_2_O_2_ (TNF1, TNF2, TNF3) and for the nanowires obtained during 6 h of the oxidation process (TNW5–TNW8) (Figure 4a,c). The obtained results are in agreement with the earlier reports concerning the effects of the micro- and nanoscale material surface topography on various types of cells [55,56,57,58]. According to these data, the nanoscale topography provides greater support for the proliferation of bone cells. This can be easily explained by the advantageous interaction between nanosize irregularities on the material surface and cells; this means improved osteoblast adhesion and spreading. Simultaneously, the results of the MTT assays carried out for the titania nanoneedles’ (TNNs) surface showed that proliferation observed after 5 days was comparable or lower than in the case of Ti6Al4V. The micro-roughness was responsible for the decreasing biointegration activity and for excluding the TNNs from the group of potential implant coatings. The microscale irregularities were also noticed to hamper cell spreading, and thus slow down cell proliferation [56,59]. In accordance with this, Rosa and Beloti, who compared the influence of various submicron- and microscale roughnesses of titanium substrates on human bone marrow cell growth, found that cell adhesion and proliferation decreased with increasing the material surface roughness [60].

Analysis of the SEM images presented in Figure 5 showed that the fibroblast cells attached well to the surface of TNF1 and TNW5 after 72 h, and particularly after 5 days. It can also be clearly seen that the number of cells attached to the surface of the nanolayers increases together with the duration of the incubation time. This phenomenon was observed for both the tested nanolayers. Fibroblasts were also well spread, covering most of the areas of the nanolayer surface and exhibited elongated shapes, especially after the 5 days incubation time. Moreover, the cells formed filopodia, which attached the cells to the surface of the arrays, penetrating deep into the nanolayers (grey arrows in Figure 5), and fibroblasts also formed filopodia among themselves (white arrows in Figure 5). Cell filopodia have been shown to “sense” biomaterial surface topographies, and play an important role in cell movement and attachment [61,62] and act as a partial regulator of cell adhesion, proliferation, and cell-cell interactions [63]. These findings are important since fibroblasts are considered to be the most common cells in connective tissue, one of the main components of peri-implant soft tissue, which is key to the formation of the peri-implant mucosal seal and helps to prevent epithelial ingrowths [64]. 

From a microbiological point of view, titanium and titanium alloy implants are described as quite resistant to microbial colonization, and are even more potent against *S. aureus* biofilm formation than cobalt and chrome implants, which are recently becoming popular in spinal surgery [65,66]. Nevertheless, bacterial biofilms on titanium surfaces can also develop, such as those formed by the oral cavity microbiome on dental implants frequently leading to peri-implant diseases [67]. Therefore, some modifications of Ti surfaces are proposed to decrease the extent of their colonization by microorganisms. Liu et al. [67] demonstrated that *Streptococcus mutans* and *Porphyromonas gingivalis* clusters formed on copper-bearing titanium alloy (Ti-Cu), fabricated by Cu immobilization into titanium, were smaller and less dense than that on commercially pure titanium. The viability of the *S. mutans* cells present on the Ti-Cu alloy was also significantly decreased [67]. According to Mathew et al. [65], plasma-spray deposition of hydroxyapatite on Ti created a surface almost 32 times less vulnerable to *S. aureus* colonization than the reference titanium. Our results were not so spectacular, but it is worth noting that the nanostructural modifications of titania alloy surfaces intended to improve their tissue integration (which was proven for mouse fibroblasts L929; Figure 5), led us to obtain porous surfaces more favorable also for microbial colonization. Ercan et al. [68] demonstrated stronger initial attachment of *S. aureus* (after 1 h of coincubation) to nanotubular Ti samples compared to conventional Ti, which increased biofilm formation on the modified surface up to 8 h. A similar observation was made previously by Puckett et al. [69], in the case of nanotubular and nanorough Ti created through anodization, while nanorough Ti surfaces created through electron beam evaporation were able to decrease the adhesion of both Gram-positive (*S. aureus*, *S. epidermidis*) and Gram-negative (*Pseudomonas aeruginosa*) bacteria. Our previous report [70] confirms the possibility of decreasing *S. aureus* adhesion by tailoring the nanotube parameters (morphology and structure). Therefore, it is necessary to have a balance between surface biocompatibility and its vulnerability to microbial adhesion and biofilm formation. It seems that proper, well-balanced parameters have been obtained for TNF2–TNF4 and TNF6. In contrast, adverse properties characterized TNF1 and TNF5. The features of the TNF1 sample changed in the successive series of the experiments, which is confirmed by the very high standard deviation (SD) values (Figure 6a). This indicates the lack of stability of the biomaterial during storage, whereas the TNF5 layer was colonized by *S*. *aureus* to a significantly greater degree than Ti6Al4V. The average percentage of staphylococcal aggregates/biofilm formation on TNF5 achieved 133.1 ± 3.1% (*p* = 0.0008) and 196.8 ± 31.2% (*p* = 0.0008) in AB and L/D, respectively, (Figure 6a). In the case of the nanowire coatings, the balance between tissue biocompatibility and the vulnerability to microbial colonization, which was noticed for the reference sample, was preserved for TNW3, TNW5, and TNW6. However, from a practical point of view (the use of the tested titania samples in implantology), TNW4, TNW7, and TNW8 coatings are the most attractive, as they demonstrated the ability to inhibit bacterial biofilm formation when assessed by L/D (Figure 6b).

In summary, the results of our work provide knowledge that the conditions for performing chemical or thermal oxidation of titanium alloys affect not only the topography of the nanostructural surfaces but also their properties that are important for the contacts with host cells and microorganisms. Among the produced titania coatings the following samples: TNF6, TNW4, TNW7, and TNW8, exhibited the optimal characteristics of hydrophilicity, biocompatibility, and antimicrobial activity, and were definitely better than that of Ti6Al4V. These features suggest their potential usefulness in the modification of titanium and titanium alloy implants. 

## 4. Materials and Methods 

### 4.1. Ti6Al4V Substrates

Titanium alloy foils (Ti6Al4V, grade 5) were cut into 10 × 10 mm and 5 × 5 mm pieces, which were ultrasonically cleaned sequentially in acetone (15 min), 80% ethanol (5 min), and deionised water (15 min). The Ti6Al4V implants used in maxillo-facial surgery have been prepared in accordance with the procedure described above. In the next step, the obtained substrates were dried in an Argon stream at room temperature. The surface of the substrates were prepared for the chemical oxidation processes in the presence of 30% H_2_O_2_ solution, and were chemically etched in a 1:4:5 mixture of HF:HNO_3_:H_2_O for 30 s, cleaned with deionised water, and dried in Argon stream. The surface of the substrates used in the thermal oxidation process was activated by its immersion in a 1:1 mixture of 36.5% HCl and water at 80 °C for 30 min. After the activation, the samples were cleaned with deionised water and dried in Argon stream. Before biological experiments, all substrates covered by titanium dioxide layers were sterilized in a steam autoclave at 120 °C and 16 psi for 30 min. 

### 4.2. Chemical Oxidation

The Ti6Al4V substrates were immersed in 50 cm^3^ 30% H_2_O_2_ solution or 30% H_2_O_2_/CaCl_2_ solution as the oxidizing agents in a round bottom flask under reflux. The H_2_O_2_/CaCl_2_ solution was prepared by the dissolution of 55.5 mg CaCl_2_ in 50 cm^3^ of 30% H_2_O_2_. The oxidation processes were carried out at 80 and 100 °C for different oxidation times, i.e., *t* = 6, 8, 10, and 12 h. The formed fibrous coatings were ultrasonically cleaned in acetone (15 min), in deionised water (15 min) and dried in Argon stream. On the basis of the preliminary SEM studies of the coatings, the following samples were chosen for the biological experiments: H_2_O_2_ (temperature [°C], time of process [h]): 80, 8 (TNF1), 100, 8 (TNF2), and 100, 12 (TNF3), and H_2_O_2_/CaCl_2_ (temperature [°C], time of process [h]): 80, 8 (TNF4), 100, 8 (TNF5), and 100, 12 (TNF6).

### 4.3. Thermal Oxidation

The thermal oxidation was conducted in a vacuum tube furnace at temperatures between 475 and 500 °C under a pressure of 1–1.5 hPa for different oxidation times, i.e., *t* = 2 h and 6 h. The oxidation process was carried out in the presence of argon or argon passed over the H_2_O_2_ surface (Ar/H_2_O_2_), using the following flow rates: 30 cm^3^/min and 100 cm^3^/min. On the basis of the preliminary SEM studies of the coatings, samples were obtained at the following conditions: Ar (flow rate [cm^3^/min], temperature [°C], time of process [h]): 30, 500, 2 (TNN1), 100, 500, 2 (TNN2), 30, 475, 2 (TNN3), 100, 475, 2 (TNN4), 30, 500, 6 (TNN5), 100, 500, 6 (TNN6), 30, 475, 6 (TNN7), and 100, 475, 6 (TNN8), and Ar/H_2_O_2_ (flow rate [cm^3^/min], temperature [°C], time of process [h]): 30, 500, 2 (TNW1), 100, 500, 2 (TNW2), 30, 475, 2 (TNW3), 100, 475, 2 (TNW4), 30, 500, 6 (TNW5), 100, 500, 6 (TNW6), 30, 475, 6 (TNW7), and 100, 475, 6 (TNW8), and were used in immunological experiments. In the studies of the antibacterial properties, the following samples were used: Ar/H_2_O_2_ (flow rate [cm^3^/min], temperature [°C], time of process [h]): 30, 500, 2 (TNW1), 100, 500, 2 (TNW2), 30, 475, 2 (TNW3), 100, 475, 2 (TNW4), 30, 500, 6 (TNW5), 100, 500, 6 (TNW6), 30, 475, 6 (TNW7), and 100, 475, 6 (TNW8), and TNNs were excluded from further studies because of their poor biocompatibility. 

### 4.4. Characterization of Titania Coatings

The structure of the produced titania based nanomaterials was characterized using X-ray diffraction (PANalytical X’Pert Pro MPD X-ray diffractometer using Cu-K_α_ radiation, grazing incidence angle mode–GIXRD; the incidence angle was equal to 1 deg), and Raman spectroscopy (RamanMicro 200 Perkin Elmer). The morphology of the produced coatings was studied using Quanta field-emission gun scanning electron microscopy (SEM; Schottky FEG). The wettability of the TNF, TNN, and TNW layers was investigated using a Krüss system for the contact angle measurement. Ten μL of distilled water were slowly deposited on the surface of analysed coatings using a screw syringe. The image was recorded and the contact angles were estimated by numerically fitting to the droplet image. The value of the contact angle for each biomaterial is the average value of five measurements.

### 4.5. Adhesion and Proliferation of L929 Cells on TNFs, TNNs, and TNWs Arrays

Murine fibroblast cell line L929 (American Type Culture Collection) culture conditions were the same as we described previously [60]. The effect of TNFs, TNNs, and TNWs on the L929 cells’ adhesion (after 24 h) and proliferation (after 72 h and 5 days) was assessed using the MTT assay (3-(4,5-dimethylthiazole-2-yl)-2,5-diphenyl tetrazolium bromide; Sigma Aldrich; Darmstadt, Germany). Briefly, after the selected incubation time, the TNF, TNN, and TNW arrays were washed gently three times with phosphate buffered saline (PBS) and transferred to a new 24-well culture plate. The MTT (5 mg/cm^3^; Sigma-Aldrich, Darmstadt, Germany) solution in RPMI 1640 medium without phenol red (Sigma-Aldrich, Darmstadt, Germany) was added to each well. After 3 h of incubation at 37 °C in a humidified atmosphere of 5% CO_2_, the solution was aspirated from each well. Then, 500 μL of dimethyl sulfoxide (DMSO; 100% *v*/*v*; Sigma Aldrich, Darmstadt, Germany) was added to each well and the plate was shaken for 10 min. The absorbance of each solution was measured at the wavelength of 570 nm with the subtraction of the 630 nm background, using a microplate reader (Synergy HT; BioTek, Winooski, VT, USA). The blank groups (TNF, TNN, and TNW arrays incubated without cells) were treated with the same procedures as the experimental groups. Culture medium without TNFs, TNNs, and TNWs served as a negative control for each experiment. All measurements were done in duplicate in three independent experiments.

### 4.6. Cell Morphology Observed by Scanning Electron Microscopy

After the selected incubation period, the samples were washed three times (for 10 min) with PBS to remove the non-adherent cells and were fixed in 2.5% glutaraldehyde (Sigma Aldrich, Darmstadt, Germany) for a minimum of 4 h (maximum of 1 week). After that, the specimens were rinsed three times with PBS for 10 min. The samples were then dehydrated in a graded series of alcohol (50%, 75%, 90%, and 100%) for 15 min, then dried in vacuum-assisted desiccators overnight and stored at room temperature until the SEM analysis was performed. The morphology of the studied nanocoatings as well as that of the adhered cells were observed using scanning electron microscopy (SEM; Quanta 3D FEG; Carl Zeiss, Göttingen, Germany).

### 4.7. Statistical Analysis in the MTT Assay

Statistical significance was determined using one-factor analysis of variance (ANOVA). As a post hoc test, the Tukey test was used. The level of significance was set at *p* < 0.05.

### 4.8. Microbial Aggregates/Biofilm Formation on the Studied Nanocoatings 

The TNF and TNW samples (size roughly 5 × 5 mm) as well as the unmodified Ti6Al4V alloy (reference sample) were exposed on *S. aureus* ATCC 29213. The samples were coincubated with bacterial suspension (OD_535_ = 0.9) for 24 h at 37 °C in stable conditions to form microbial aggregates/biofilm. Ti6Al4V samples in culture media were used as the negative control. To evaluate the staphylococcal aggregates/biofilm formation, Alamar Blue assay (AB; Invitrogen, Thermo Fisher Scientific, Eugene, OR, USA) and LIVE/DEAD BacLight Bacterial Viability kit (L/D; Invitrogen, Thermo Fisher Scientific, Eugene, OR, USA) were used after mechanical recovery of the microbial cells from the tested surfaces. Two independent sets of experiments, each in quadruplicate, were prepared.

The staining protocol for AB was used as recommended by the manufacturer. Finally, the absorbance of the bacterial suspensions was determined at 550 nm and 600 nm using the multifunctional counter Victor2 (Wallac, Turku, Finland). The percentage of AB reduction was calculated according to the manufacturer formula:

(1) $${\mathrm{AB}}_{\mathrm{reduction}}\left[\%\right]=\frac{\varepsilon_{\mathrm{OX}}\lambda_{2}\times A\lambda_{1}-\varepsilon_{\mathrm{OX}}\lambda_{1}\times A\lambda_{2}}{\varepsilon_{\mathrm{RED}}\lambda_{1}\times A^{'}\lambda_{2}-\varepsilon_{\mathrm{RED}}\lambda_{2}\times A^{'}\lambda_{1}}\times 100$$

where: _OX_—molar extinction coefficient of the AB oxidized form, _RED_—molar extinction coefficient of the AB reduced form, *A*—absorbance of the test wells, *A*′—absorbance of the negative control well, λ_1_—550 nm, λ_2_—600 nm.

The obtained results are presented as the percentage of metabolically active bacteria reclaimed from aggregates/biofilm formed on tested titanium samples in comparison to that on the unmodified Ti6Al4V alloy (control sample) considered as 100%, calculated based on the mean AB reduction.

The L/D was used as recommended by the manufacturer. The fluorescence at 485_ex_/535_em_ nm (Syto9) of the bacteria reclaimed from the aggregates/biofilm formed on the tested titanium samples was measured using the multifunctional plate reader Victor2 and was compared with the fluorescence of the bacteria from the reference sample (Ti6Al4V) to calculate the percentage of viable cells.

### 4.9. Statistical Analysis in the Microbiological Tests

All data were analysed using the STATISTICA 10.0 PL software (StatSoft Inc., Tulsa, OK, USA). The nonparametric Kruskal–Wallis one-way ANOVA was used to compare differences among the titanium samples. *p* < 0.05 was considered significant.

### 4.10. Photocatalytic Activity Studies

The photocatalytic activity of the TNFs and TNWs was studied on the basis of the photodegradation of methylene blue (MB), according to the previously described measurement procedure [71].
